# Heterologous phosphoketolase expression redirects flux towards acetate, perturbs sugar phosphate pools and increases respiratory demand in *Saccharomyces cerevisiae*

**DOI:** 10.1186/s12934-019-1072-6

**Published:** 2019-02-01

**Authors:** Alexandra Bergman, John Hellgren, Thomas Moritz, Verena Siewers, Jens Nielsen, Yun Chen

**Affiliations:** 1 Department of Biology and Biological Engineering, Chalmers University of Technology, Kemivägen 10, 41296 Gothenburg, Sweden; 2 Novo Nordisk Foundation Center for Biosustainability, Chalmers University of Technology, 41296 Gothenburg, Sweden; 3 Department of Forest Genetics and Plant Physiology, Swedish University of Agricultural Sciences, Umeå Plant Science Center (UPSC), 901 83 Umeå, Sweden; 4 Swedish Metabolomics Centre, Umeå Plant Science Center (UPSC), 901 83 Umeå, Sweden; 5 Novo Nordisk Foundation Center for Biosustainability, Technical University of Denmark, 2800 Kongens Lyngby, Denmark

**Keywords:** *Saccharomyces cerevisiae*, Phosphoketolase, Acetyl-phosphate, Acetate, Acetyl-CoA, Physiology, Sugar phosphate, RNAseq

## Abstract

**Introduction:**

Phosphoketolases (Xfpk) are a non-native group of enzymes in yeast, which can be expressed in combination with other metabolic enzymes to positively influence the yield of acetyl-CoA derived products by reducing carbon losses in the form of CO_2_. In this study, a yeast strain expressing Xfpk from *Bifidobacterium breve*, which was previously found to have a growth defect and to increase acetate production, was characterized.

**Results:**

Xfpk-expression was found to increase respiration and reduce biomass yield during glucose consumption in batch and chemostat cultivations. By cultivating yeast with or without Xfpk in bioreactors at different pHs, we show that certain aspects of the negative growth effects coupled with Xfpk-expression are likely to be explained by proton decoupling. At low pH, this manifests as a reduction in biomass yield and growth rate in the ethanol phase. Secondly, we show that intracellular sugar phosphate pools are significantly altered in the Xfpk-expressing strain. In particular a decrease of the substrates xylulose-5-phosphate and fructose-6-phosphate was detected (26% and 74% of control levels) together with an increase of the products glyceraldehyde-3-phosphate and erythrose-4-phosphate (208% and 542% of control levels), clearly verifying in vivo Xfpk enzymatic activity. Lastly, RNAseq analysis shows that Xfpk expression increases transcription of genes related to the glyoxylate cycle, the TCA cycle and respiration, while expression of genes related to ethanol and acetate formation is reduced. The physiological and transcriptional changes clearly demonstrate that a heterologous phosphoketolase flux in combination with endogenous hydrolysis of acetyl-phosphate to acetate increases the cellular demand for acetate assimilation and respiratory ATP-generation, leading to carbon losses.

**Conclusion:**

Our study shows that expression of Xfpk in yeast diverts a relatively small part of its glycolytic flux towards acetate formation, which has a significant impact on intracellular sugar phosphate levels and on cell energetics. The elevated acetate flux increases the ATP-requirement for ion homeostasis and need for respiratory assimilation, which leads to an increased production of CO_2_. A majority of the negative growth effects coupled to Xfpk expression could likely be counteracted by preventing acetate accumulation via direct channeling of acetyl-phosphate towards acetyl-CoA.

**Electronic supplementary material:**

The online version of this article (10.1186/s12934-019-1072-6) contains supplementary material, which is available to authorized users.

## Introduction

The yeast *Saccharomyces cerevisiae* is commonly used in industrial production partly due to its robustness and ease of genetic manipulation, and it has been genetically engineered to increase or enable production of a multitude of biochemicals. The biosynthesis of many industrially interesting chemicals are formed from acetyl coenzyme A (acetyl-CoA), which represents a key intermediate in metabolism [1]. The native production route of cytosolic acetyl-CoA in *S. cerevisiae* depends on the enzymatic activities of pyruvate decarboxylase (Pdc), acetaldehyde dehydrogenase (Ald) and acetyl-CoA synthase (Acs). The route is neither carbon nor energy efficient, as one molecule of CO_2_ is lost per pyruvate entering the pathway and two equivalents of ATP are consumed when acetate is activated by Acs (ATP → AMP). Thus, many alternative precursor strategies have been investigated in *S. cerevisiae* with potential to increase the theoretical yield of acetyl-CoA derived products. For example, engineering strategies based on ATP citrate lyase [2–6], acetylating acetaldehyde dehydrogenase [7], pyruvate formate lyase [7, 8], pyruvate oxidase [9], and a cytosolic pyruvate dehydrogenase [10] have all been functionally established in yeast, and can produce cytosolic acetyl-CoA without or with reduced consumption of ATP, starting from pyruvate or pyruvate-derived metabolites.

Another attractive option for generating cytosolic acetyl-CoA in *S. cerevisiae* is based on a metabolic route where the heterologous enzymes phosphoketolase (Xfpk) and phosphotransacetylase (Pta) are expressed [11–15]. Such a route can produce acetyl-CoA directly from fructose-6-phosphate and xylulose-5-phosphate (see Eqs. 1–3), and represents a synthesis route which is energy as well as carbon neutral.

1 $$ xylulose{\text{-}}5{\text{-}}phosphate \, + \, Pi \to glyceraldehyde{\text{-}}3{\text{-}}phosphate \, + \, acetyl{\text{-}}phosphate \, \left( {Xfpk} \right) $$

2 $$ fructose{\text{-}}6{\text{-}}phosphate \, + \, Pi \to erythrose{\text{-}}4{\text{-}}phosphate \, + \, acetyl{\text{-}}phosphate \, \left( {Xfpk} \right) $$

3 $$ acetyl{\text{-}}phosphate \, + \, CoA \leftrightarrow acetyl {\text{-}}CoA \, + \, P_{i} \left( {Pta} \right) $$

Among all the above-mentioned pathways, the Xfpk/Pta pathway represents the one with the highest theoretical yield of several products, such as citric acid, palmitic acid and farnesene [16], as its acetyl-CoA production step circumvents CO_2_ emission. In the case of products which require ATP for their biosynthesis, a higher yield can theoretically be obtained if using a combined pathway approach, because the Xfpk/Pta pathway generates less ATP as the energy generating proportion of glycolysis is bypassed [16]. This was experimentally demonstrated by utilizing a Xfpk from *Leuconostoc mesenteroides*, a Pta from *Clostridium kluyveri* in combination with an acetylating acetaldehyde dehydrogenase from *Dickeya zeae* to increase production of farnesene in *S. cerevisiae* by 25%, while simultaneously reducing oxygen consumption by 75% [15].

In many metabolic scenarios multiple genetic modifications are simultaneously introduced into an organism and the combined effects on cellular behavior and productivity are investigated. However, using this approach, important aspects of how specific modifications affect the host physiology might be overlooked and it complicates the procedure of optimizing the overall cell factory. Furthermore, if non-native enzymes are expressed this should be of particular high concern, as their enzymatic activity and/or resulting metabolites might influence cellular physiology in unforeseen ways.

We previously expressed a group of phosphoketolases in *S. cerevisiae* and conducted in vitro as well as in vivo tests to find candidates with high enzymatic activity. Several phosphoketolases with high enzymatic activity were identified, but it was found that expression was coupled to impaired growth of the host, both in terms of specific growth rate on glucose and ethanol and final biomass yield [14]. The expression was also found to be coupled with an increased acetate production, a phenomenon which has been attributed to promiscuous activity of the endogenous glycerol-3-phosphatases Gpp1 and Gpp2 upon acetyl-phosphate [14, 15].

A growth defect represents a significant drawback in biorefinery applications, as it is likely to reduce both yield and productivity of the process. To gain a better understanding of how phosphoketolases affects the physiology of *S. cerevisiae* host, we here quantified growth physiology, sugar phosphate levels and transcript levels in a strain of yeast expressing a phosphoketolase from *Bifidobacterium breve*. This particular enzyme has a high specific activity towards xylulose-5-phosphate as well as fructose-6-phosphate, with the ratio approximate 3:2 [14]. The higher specific activity towards fructose-6-phosphate, which is present at a higher concentration during growth on glucose [17], may have greater application in exploiting this enzyme for production of acetyl-CoA derived chemicals.

## Results and discussion

### Phosphoketolase leads to increased acetate formation and respiratory demand

As our previous study showed that Xfpk-expression was coupled to a reduced specific growth rate, longer diauxic shift and lower biomass formation in shake flask cultivations [14], we wanted to examine a phosphoketolase expressing strain in greater detail to understand the mechanisms behind its altered physiology. We did so by conducting controlled bioreactor cultivations of yeast harboring either an empty plasmid—referred to as control; or yeast harboring a plasmid encoding a highly active enzyme from *B. breve*—a strain referred to as xfpk(BB). The strains were initially cultivated in batch mode with 2% glucose at pH 5, and when the culture reached the ethanol phase, glucose-limited chemostat mode was initiated with a dilution rate of D = 0.1 h^−1^ and a glucose concentration in the feed of 7.5 g L^−1^.

As shown in Table 1, the maximum specific growth rate of xfpk(BB) was reduced approximately 20% compared with the control during the exponential batch phase, while the biomass yield was reduced roughly 10%. In contrast to a decrease in most specific rates calculated during the batch phase, however, the rates of acetate production and oxygen consumption were significantly higher than the control (Table 1). The higher rate of acetate formation indirectly implies that phosphoketolase enzyme activity is present, as the produced acetyl-phosphate (AcP) can be degraded to acetate by nonspecific phosphatases Gpp1 and Gpp2 [14]. The observed increase in oxygen consumption rate could indicate that the demand for respiration and energy was enhanced for the xfpk(BB) strain.

**Table 1:** Physiological parameters calculated for control and strain xfpk(BB) in batch and chemostat mode

Strain	Batch		Chemostat	
	Control	xfpk(BB)	Control	xfpk(BB)
µ(max) (h^−1^)	0.353 ± 0.008	0.266 ± 0.006	0.104 ± 0.002	0.103 ± 0.003
Y(x/s) (gCDW/gGlucose)	0.135 ± 0.00	0.123 ± 0.00	0.479 ± 0.01	0.409 ± 0.01
q(Glucose) (mmol gCDW^−1^ h^−1^)	− 14.42 ± 0.36	− 12.19 ± 0.39	− 1.18 ± 0.06	− 1.36 ± 0.03
q(EtOH) (mmol gCDW^−1^ h^−1^)	21.46 ± 0.75	17.26 ± 0.52	–	–
q(Acetate) (mmol gCDW^−1^ h^−1^)	0.61 ± 0.04	1.38 ± 0.03	–	–
q(Glycerol) (mmol gCDW^−1^ h^−1^)	1.13 ± 0.03	0.62 ± 0.04	–	–
q(Pyruvate) (mmol gCDW^−1^ h^−1^)	0.13 ± 0.01	0.15 ± 0.00	–	–
q(Succinate) (mmol gCDW^−1^ h^−1^)	0.02 ± 0.0	0.01 ± 0.0	–	–
q(CO_2_) (mmol gCDW^−1^ h^−1^)	30.01 ± 0.32	28.16 ± 0.97	2.91 ± 0.19	3.34 ± 0.09
q(Biomass) (mmol gCDW^−1^ h^−1^)	14.37 ± 0.31	10.81 ± 0.25	4.13 ± 0.31	4.07 ± 0.25
q(O_2_) (mmol gCDW^−1^ h^−1^)	− 5.49 ± 0.53	− 7.63 ± 0.76	− 2.61 ± 0.20	− 3.35 ± 0.12
Carbon balance	107% ± 2%	107% ± 2%	99% ± 3%	92% ± 1%

Strains were grown in biological quadruplicates in minimal media with 2% glucose during batch phase and 0.75% glucose in the feed in chemostat conditions. Values shown correspond to averages ± standard deviation

**Fig. 1:**
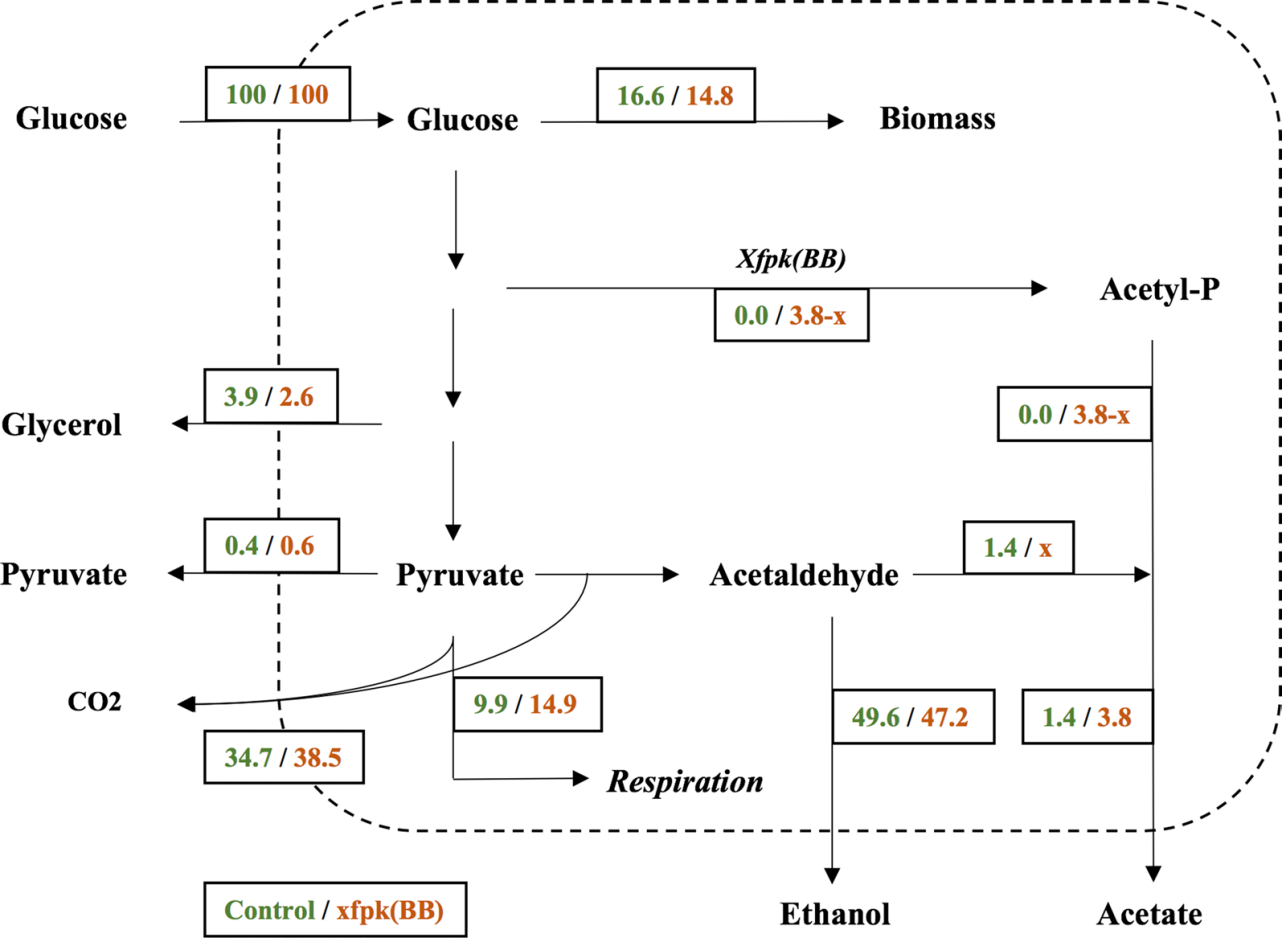
Carbon fluxes (in % C-moles) of exponentially growing strains normalized to the glucose uptake rate. Flux towards respiration is estimated as the difference between flux towards CO_2_ and half the flux towards ethanol. Fluxes corresponding to below 0.1% of the total flux are not shown in the figure. The strains were grown in biological quadruplicates in minimal media with 2% glucose at pH 5

To further visualize how phosphoketolase expression affects carbon distribution, all rates during the batch phase (Table 1) were normalized to the specific glucose uptake rate and a schematic figure of how normalized carbon fluxes (expressed in C-moles) were distributed within the two strains during exponential phase is shown in Fig. 1. Acetate flux was most significantly increased, up to about 3.8% of the total carbon flux, an approximate threefold increase compared with the control value of 1.4%. While the fluxes towards the most common fermentation products, such as glycerol and ethanol were decreased, the total flux towards CO_2_ increased by approximately 10% in the xfpk(BB) strain compared with the control. If the carbon flux towards respiration was estimated as the difference between the total CO_2_ flux and the CO_2_ produced via pyruvate decarboxylation in the cytosol (i.e. half of the C-mol flux of ethanol), the respiratory demand increased with approximately 50% in xfpk(BB) compared with the control. This is well supported by an observed increase in O_2_ consumption, where q(O_2_) was calculated to be 5.5 mmol gCDW^−1^ h^−1^ for the control and 7.6 mmol gCDW^−1^ h^−1^ for xfpk(BB).

In chemostat mode, although the sugar metabolism of both strains was completely respiratory, the biomass yield on glucose of xfpk(BB) was 15% less than the control. This lower biomass yield was in agreement with higher rates of oxygen consumption and CO_2_ production during chemostat conditions (Table 1). The consequence of Xfpk expression is a channeling of carbon flux from glycolysis towards acetate without any energy formation. Acetate has to be activated by acetyl-CoA synthetase using two moles ATP invested per mole of acetate after which it is consumed via respiratory processes. Thus, the reduction in energy efficiency due to the heterologous phosphoketolase flux may explain the lower biomass yield in xfpk(BB).

The rate of O_2_ consumption and CO_2_ production for the control strain (q(O_2_) = 2.61 ± 0.20 and q(CO_2_) = 2.91 ± 0.19 mmol gCDW^−1^ h^−1^) is in good agreement with a previous chemostat study performed using the same conditions (q(O_2_) = 2.74 ± 0.03 and q(CO_2_) = 2.85 ± 0.04 mmol gCDW^−1^ h^−1^) [18]. The same study also conducted a cultivation using acetate as the limiting nutrient, increasing CO_2_ and O_2_ fluxes to more than 260% of the control (q(O_2_) = 7.4 ± 0.23 and q(CO_2_) = 7.45 ± 0.18 mmol gCDW^−1^ h^−1^). Using these data, strain xfpk(BB) could be estimated to represent a case where the substrate source is a mixture of glucose and acetate, and the proportion of each substrate is a linear combination of the gas flux under glucose and acetate conditions (2.8x + 7.4y = 3.35; x + y = 1). This corresponds to a situation where approximately 12% of the carbon available in glucose is converted into acetate by the phosphoketolase in glucose-limited conditions. This number would be estimated to 2.4% for batch conditions if simply considering the difference in acetate flux between control and strain xfpk(BB) (Fig. 1). As acetate produced by Xfpk in the chemostat represents an initial loss of ATP when glucose is activated by hexokinase (compared to the situation were acetate is provided as substrate directly), the calculated value of 12% could theoretically be even larger. A higher flux through the phosphoketolase at chemostat conditions compared to batch cultivations could be due to the fact that carbon limitation allows for efficient co-consumption of glucose and acetate, which prevents accumulation of acetate.

### Proton decoupling contributes to the growth defect of a phosphoketolase-expressing strain

As discussed above, based on the normalized carbon fluxes, it is likely that at least 2.4% of all carbon entering the cell is shuttled through the phosphoketolase towards acetyl-phosphate during glucose phase, and from there reaching the state of acetate via the actions of unspecific phosphatases Gpp1 and Gpp2 [14]. This is a relatively small number, which, as shown above, however has severe consequences on cell physiology.

When acetate is produced in the cytosol, it dissociates due to the neutral pH and causes an acidification of the cytoplasm. Thus, the cell is required to keep its intracellular pH and prevent accumulation of intracellular acetate ions by pumping out both protons and anions using ATP-dependent processes [19]. Acetate can traverse the cell membrane in its lipophilic HA-form (the predominant species if the pH is below its pK_a_ = 4.76) and thus re-enter the cell, creating a futile cycle referred to as proton decoupling. By this process, higher acetate production rates lead to an increased ATP-demand, which is likely to result in a higher respiratory demand (oxygen consumption).

**Fig. 2:**
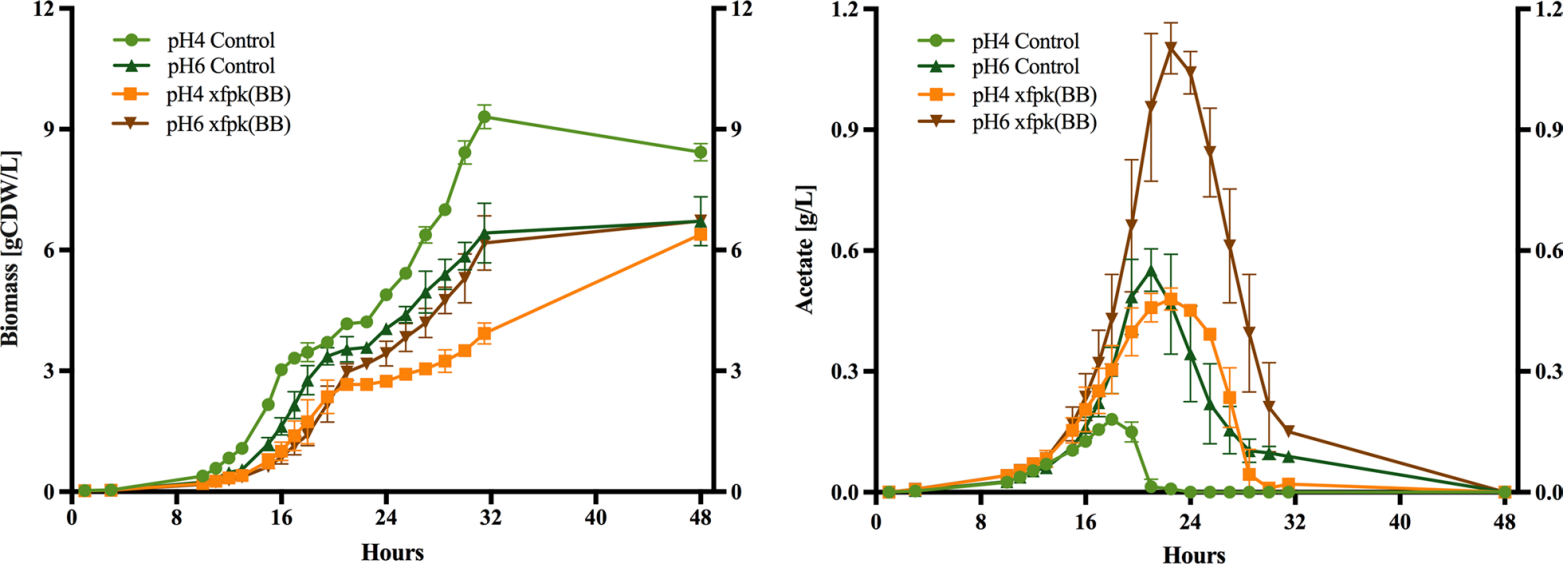
Biomass and acetate accumulation of control and phosphoketolase expressing strain [xfpk(BB)]. Strains were cultivated in biological duplicates in pH-controlled (pH 4 and pH 6) batch cultivations in minimal media with 2% glucose. Error bars are ± standard deviation

In order to determine the influence of proton decoupling of the produced acetate on the negative growth characteristics seen in strain xfpk(BB), we set out to investigate the growth profiles of both strains at pH 4 and 6 at which the HA-form of acetic acid should be present at 85% versus 5%. pH-controlled batch cultivations were conducted in biological duplicates with 2% glucose. An overview of cell and acetate accumulation profiles can be found in Fig. 2 and a detailed physiological characterization during the exponential batch phase is summarized in Additional file 1: Table S1. For both strains, the maximum acetate concentrations obtained at pH 6 were about two to threefold of those seen at pH 4 (Fig. 2, right graph), which agreed with previous study that acetate production was influenced directly by environmental conditions [20]. Under both pH conditions, acetate accumulation in xfpk(BB) was more than twofold higher than the control. This was consistent with an increase in the CO_2_-production rate for xfpk(BB) compared to the control at both pHs.

During the exponential growth on glucose, an increased pH seems to have neglectable effect on cell growth and biomass yield for the control strain, whereas at pH 6, the biomass yield significantly increases for xfpk(BB) compared to its pH 4 counterpart, from 89% to about 95% of that of the control in glucose phase—indicating a more energy efficient growth. In both strains, glycerol excretion increases with increasing pH, while ethanol formation decreased and CO_2_ formation remained constant (Additional file 1: Table S1). The decrease in ethanol flux and stable CO_2_ production rates seen for both strains indicated that more CO_2_ was produced through respiratory processes at pH 6. Indeed, previous studies of yeast physiology in response to different pH support that a high pH stimulates TCA cycle activity [20]. The oxygen consumption rate is increased for the control strain at pH 6, but due to a failure of a O_2_-detection unit, only one value for oxygen consumption for xfpk(BB) at pH 6 was obtained, which does not show an increase in oxygen consumption for xfpk(BB) compared to pH 4.

If an increased respiratory demand of xfpk(BB) during the glucose consumption phase could be fully explained by a larger ATP demand due to proton decoupling, the CO_2_ produced from respiratory processes (estimated as q(CO_2_)-q(EtOH)) would only be moderately larger than the control for xfpk(BB) at pH 6. This number is 12.39 mmol gCDW^−1^ h^−1^ for the control and 18.00 mmol gCDW^−1^ h^−1^ xfpk(BB), which is larger than the difference seen at pH 4 (8.90 and 12.26 mmol gCDW^−1^ h^−1^, respectively). This could be an indication that there is a degree of co-consumption of acetate during growth on glucose, which should be exclusively respiratory. Co-consumption of glucose and acetate is not considered to be typical for *S. cerevisiae*, but it has been observed previously for CEN.PK yeast strains [21].

After glucose was depleted from the media, an increase in pH reduced the biomass yield of the control, resulting in a final biomass yield drop to about 80% compared with growth at pH 4. At the same time, xfpk(BB) grew faster at pH 6 than that at pH 4, reaching a growth rate almost equal to the control at pH 6, with a concurrent significant biomass yield increase (see Fig. 2, left graph, and Additional file 1: Table S1). During this phase, acetate is consumed by both strains and depending on the amount of acetate entering the cell, acetate might just be metabolized (simulated at pH 6) or required to be actively pumped out alongside protons during the consumption of acetate in order to keep cellular pH homeostasis (simulated at pH 4). When the produced acetate levels were low, as for the control at pH 4, proton decoupling would not have a major impact, as the compound could be quickly consumed (within 4 h for the control). The maximum extracellular concentration of acetate observed for the control (0.15 g L^−1^) was reached at an earlier stage by xfpk(BB) due to an approximate fourfold higher production rate (Fig. 2, right graph; Additional file 1: Table S1). In addition, with the maximum concentration of acetate being threefold higher than that of the control (Fig. 2, right graph), xfpk(BB) was required to handle acetate-mediated decoupling for a prolonged period of time (closer to 10 h) as well as at higher level. At pH 6, higher production rates and maximum levels of acetate were observed for both strains, but lack of any growth rate difference between the strains in the ethanol phase indicated that the uncoupling effect of the acid was small.

The positive effects of an increased pH observed for strain xfpk(BB) in terms of biomass yield and an increased growth rate on non-fermentable carbon sources, suggest that proton decoupling influenced the physiology of strain xfpk(BB) at lower pH, and that an increased pH partly reduced the acetate-imposed ATP-burden related to ionic transport.

### Phosphoketolase expression significantly alters sugar phosphates levels

As the direct substrates and products of the enzyme phosphoketolase are sugar phosphates, we set out to investigate if its expression leads to altered intracellular levels, which in turn could give additional insight to how this influences the cell physiology. We quantified the sugar phosphates glucose-1-phosphate (G1P), glucose-6-phosphate (G6P), fructose-6-phosphate (F6P), dihydroxyacetone phosphate (DHAP), glyceraldehyde-3-phosphate (GAP), 3-phosphoglyceric acid (3PGA), 2-phosphoglyceric acid (2PGA), ribulose-5-phosphate (Ru5P), ribose-5-phosphate (R5P), xylulose-5-phosphate (X5P), erythrose-4-phosphate (E4P) and sedoheptulose-7-phosphate (S7P).

**Fig. 3:**
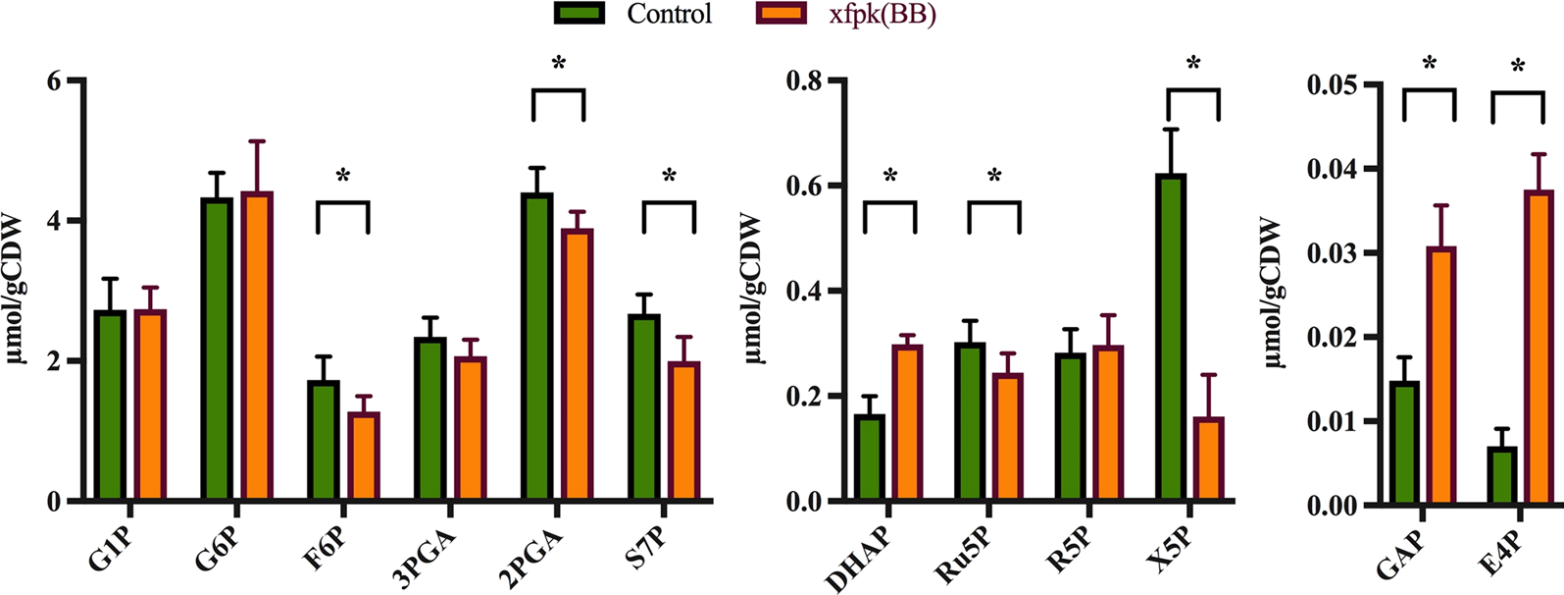
Quantification of sugar phosphates in the control strain and xfpk(BB) grown in glucose-limited chemostats (D = 0.1). Strains were cultivated in biological quadruplicates. Significant changes are indicated with asterisks (*p < 0.05 and **p < 0.001; Students t-test, two-sided, unequal variance assumed), error bars equal ± standard deviation

The sugar phosphate levels from chemostat cultivations [where the specific growth rate was the same for both strains, but the glucose uptake rate was higher for xfpk(BB)] are shown in Fig. 3 and results from batch cultivations [where the specific growth rate and glucose uptake rate were lower for xfpk(BB)] are shown in Additional file 1: Figure S1. The quantified concentrations in the control strains are in good agreement with previous reports [22], indicating that the measurements are reliable. The observed changes in sugar phosphate concentrations associated with phosphoketolase expression were found to be the same for batch and chemostat cultivations. Even though the glucose uptake rate was about tenfold lower in the chemostat versus the batch cultivation, the intracellular concentrations only decreased about twofold.

Significant changes in sugar phosphate concentrations can be seen for all expected phosphoketolase substrates and products (F6P, X5P, E4P, GAP) but also for some metabolic species which lie in close proximity to the substrates/products within the metabolic network (Ru5P, S7P, DHAP) (Fig. 3). We previously showed that Xfpk from *Bifidobacterium breve* has activity towards X5P as well as F6P, with a ratio of approximate 3:2 [14]. This is supported in this study, as the concentrations of both X5P and F6P were significantly decreased in xfpk(BB) compared with the control. While X5P levels were reduced about 75% compared with the control and GAP levels increased about twofold, F6P concentration decreased 25% and E4P concentration increased by 540% compared with the control. The dramatic increase in E4P would likely be useful in metabolic engineering applications where aromatic amino acids or chemicals are of interest, as E4P is reported to be a limiting factor in their biosynthesis [23]. It should be noted that there is a large gap between the molar difference of consumed substrate and formed product, as the molar increase in GAP or E4P is much lower than the reduction in X5P or F6P, suggesting strong thermodynamic driving forces converting the enhanced products onwards. For example, GAP is isomerised to DHAP by triose phosphate isomerase (Tpi1)—a reaction with an equilibrium ratio of about 1:20 [24]—which is in agreement with a twofold increase of DHAP concentration seen when phosphoketolase was expressed. The common product of the two phosphoketolase catalyzed reactions is acetyl-phosphate, which unfortunately could not be quantified with our method, but its continuous formation was indirectly measured as enhanced levels of acetate in the batch phase (Fig. 2).

In addition to phosphoketolase-connected substrates and products, Ru5P, 2PGA and S7P were also significantly decreased in xfpk(BB) compared with the control. Ru5P is the precursor of X5P, and was therefore probably reduced when X5P was consumed by a new reaction. S7P is involved in several carbon-rearrangement reactions within the pentose phosphate pathway, for example in the conversion of R5P + X5P ⟷ S7P + GAP by transketolase (e.g. Tkl1) and S7P + GAP ⟷ F6P + E4P by transaldolase (e.g. Tal1), and this could explain the decreased level of this metabolite.

Altered intracellular levels of metabolites could potentially cause perturbations of the rate of glycolytic and pentose phosphate pathway flux as they cause changes in the thermodynamic equilibriums and substrate pools of enzymes. For example, the twofold increase in DHAP and GAP-levels measured would theoretically require a fourfold increase in fructose-1,6-bisphosphate (F-1,6-BP) levels if the thermodynamic equilibrium state of the wild type should be maintained (K = [DHAP][GAP]/[F-1,6-BP]) [24], which is improbable as the level of F6P—the precursor of F-1,6-BP—simultaneously decreased.

### Phosphoketolase expression leads to a major transcriptional reprogramming around acetate formation and metabolism

**Fig. 4:**
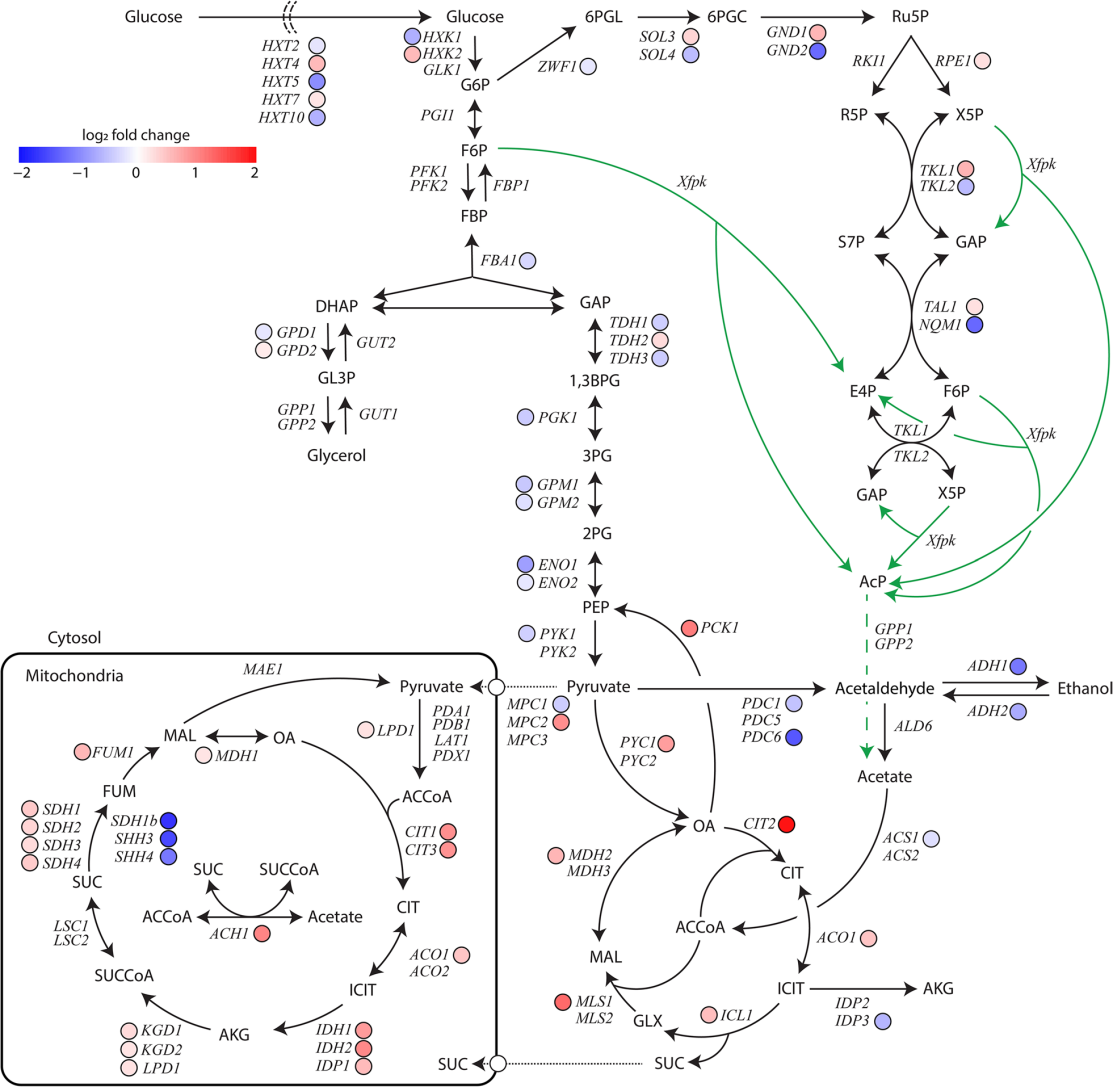
Transcriptional responses to phosphoketolase expression of central carbon metabolism genes expressed as log_2_-fold changes (padj < 0.01). Extent and type (down/up) of the fold change is indicated by shading of blue (down) and red (up) color. Samples were taken from glucose-limited chemostats in biological quadruplicates

As phosphoketolase expression resulted in significant changes in cell physiology and sugar phosphate levels, we further wanted to get a cell-wide view of how phosphoketolase expression influences the host strain. We therefore conducted RNAseq-analysis of the chemostat-cultivated strains to map all transcriptional changes imposed by phosphoketolase expression. A PCA plot (Additional file 1: Figure S2) shows that the quadruplicates of the different strains group together and are well separated along the first principle component (explaining 92% of the variance), which shows high quality of the data. The differential gene expression analysis shows that there are 2024 differentially expressed genes (DEG) with an adjusted p-value smaller than 0.01 and absolute log_2_ fold change (|LCF|) larger than 0.1. From this group, DEGs with a |LCF| > 0.5, |LCF| > 1.0 and |LCF| > 1.5 equalled 447, 120 and 47, respectively. In order to get a broader view of differential expression patterns compared to simply looking at large |LCF|-values where distinct but weak trends among gene-sets might be masked, a summary of genes involved in the central carbon metabolism with a padj < 0.01 are-summarized in Fig. 4. Furthermore, a heatmap produced from gene-set analysis with PIANO is shown in Additional file 1: Figure S3.

The majority of the glycolytic genes were weakly downregulated in xfpk(BB) compared with the control. Genes *PDC1* and *PDC6*, which are responsible for pyruvate decarboxylation, and *ADH1* and *ADH2*, whose gene products catalyze ethanol formation and degradation, respectively, are moderately to strongly downregulated. This combined set of downregulations could possibly represent a carbon redistribution in xfpk(BB) as part of the glucose is shuttled through the phosphoketolase (Fig. 4), resulting in enhanced flux towards acetate. As the strains were having full respiratory metabolism, this upregulated level of acetate formation would require a higher capacity of the glyoxylate cycle and/or the TCA cycle.

The first set of genes involved in consumption of acetate are *ACS1* and *ACS2*, which catalyze the activation of acetate to acetyl-CoA while simultaneously converting ATP to AMP. *ACS1* expression is repressed by glucose but completely de-repressed in glucose-limited conditions such as a chemostat, while *ASC2* is reported to be constitutively expressed [25]. There was no detected upregulation of either *ACS1* or *ACS2*; however, as no extracellular acetate was detected for xfpk(BB), the activation of acetate does not appear to be limiting in the chemostat.

Consistent with the Xfpk-carbon redistribution, some of the most significant transcriptional changes in strain xfpk(BB) were in upregulation of genes involved in the glyoxylate and TCA cycle (Fig. 4), which are essential for growth on non-fermentable carbon sources. Within the glyoxylate cycle, the genes *CIT2*, *ACO1*, *ICL1*, *MLS1* and *MDH2* were upregulated. Especially, the strong upregulation of *CIT2* and *MLS1* could indicate an increased level of condensation of extra-mitochondrial acetyl-CoA derived from acetate. In connection with the upregulation of the glyoxylate cycle, both *PCK1* and *PYC1*, which catalyze the conversion of oxaloacetate (OA) to phosphoenolpyruvate (PEP) and conversion of pyruvate to OA, respectively, were upregulated. These two reactions are essential steps in the gluconeogenesis pathway required for consumption of non-glucose carbon sources like ethanol or acetate [18, 26]. An increased ability to convert pyruvate to OA possibly serves as a response to an increased glyoxylate cycle flux, as OA is a required co-substrate in the initial condensation step with acetyl-CoA catalyzed by Cit2.

Within the TCA-cycle, the majority of the genes were upregulated (*LPD1*, *CIT1*, *CIT3*, *ACO1*, *IDH1*, *IDH2*, *IDP1*, *KGD1*, *KGD2*, *SDH1*, *SDH2*, *SDH3*, *SDH4*, *FUM1*, *MDH1*), while some remain unaffected (*PDA1*, *PDB1*, *LAT1*, *PDX1*, *LSC1*, *LCS2*, *MAE1*). There were a few genes related to the succinate dehydrogenase reaction being strongly downregulated (*SDH1b*, *SHH3*, *SHH4*). However, as Shh3 and Shh4 only are putative iso-enzymes to Sdh1-4 and their basal expression was lower compared to the other *SDH*-genes, they are unlikely to have high influence on succinate conversion rates. Furthermore, *SDH1b* is not considered to have an important role in mitochondrial respiration [27]. *ACH1* encoding a mitochondrial CoA-transferase important for growth on acetate [28, 29], was significantly upregulated in the xfpk(BB) strain, which also correlates with an increased demand of acetate degradation. When performing gene set analysis (Additional file 1: Figure S3), mitochondrial translation, ATP synthesis coupled transport, cristae formation, mitochondrial respiratory chain complex IV assembly and aerobic respiration processes were all distinctly upregulated. Taken together, these transcriptional changes clearly support that xfpk(BB) had an increased demand of respiratory activity and ATP-synthesis, which is likely to be due to a more energy inefficient route for acetate production and a requirement for metabolizing the increased amounts of acetate via respiration.

Transcription of transporters required for intracellular transport of acetyl-CoA over the peroxisomal and mitochondrial membranes were also affected by Xfpk-expression. For example, genes related to acetyl-CoA/carnitine transport across intracellular membranes (*CRC1*, *YAT1*, *YAT2* and *CAT2*) were all moderately to strongly upregulated in xfpk(BB), indicating the increased need to metabolize acetate. However, as *S. cerevisiae* lacks an endogenous route for l-carnitine biosynthesis [30] and the cells are grown in minimal media, they are not able to utilize this transport route.

Genes within the pentose phosphate pathway did not appear to be regulated in a distinct way. Thus, for genes encoding iso-enzymes for certain catalytic steps one gene was found to be upregulated whereas the other was downregulated (e.g. *SOL3*/*4*; *GND1*/*2*; *TKL1*/*2*; *TAL1*/*NQM1*). A microarray study of acetate-limited conditions has previously been shown to significantly downregulate expression of the gene pairs *SOL3/4* and *GND1/2* as well as *TKL2* in comparison to glucose grown cells [18]. This indicates that an increased acetate production only partially was responsible for the differential expression patterns seen in this study. The altered levels of sugar phosphates within the PPP could possibly also play a role in affecting expression, but studies investigating the transcriptional regulation with respect to metabolite levels are lacking to support this.

An interesting observation of the RNAseq analysis is that expression of multiple genes related to uracil-biosynthesis were highly upregulated while the gene *URA3* was strongly downregulated in xfpk(BB). The host strain is auxotrophic for uracil, and a copy of the native *URA3*-gene is expressed from the 2µ-plasmid to ensure vector-retention during cultivation in minimal media. Thus, it appears as if the cell actively keeps the plasmid present at a low-copy number in order to minimize the damage of the heterologous phosphoketolase. Indeed, when including the xfpk(BB) sequence in the genome sequence to which the RNAseq reads are aligned to, it shows that xfpk(BB) was expressed at levels lower than the 60 most highly expressed yeast-genes even though expressed from the *TEF1* promoter on a multicopy plasmid, while the native *TEF1* gene was expressed among the top 20 genes. This suggest that a higher flux through the enzyme could be possible if expressed in a way that ensures that toxicity induced by acetate is minimized.

## Conclusions

Our results clearly show that the major physiological alterations resulting from expression of the phosphoketolase in *S. cerevisiae* are effects related to the native hydrolysis of the phosphoketolase product acetyl-phosphate to acetate. This forces the cell to spend energy on maintaining intracellular ion balances, in particular at low pH, as indicated by the controlled pH-cultivations. Furthermore, the energy expenditure during acetate consumption is increased, as the net cost for metabolizing each mole of acetic acid produced by the phosphoketolase is 2 ATP due to the loss of ATP generation in glycolysis, while for the control it will be 1 ATP per each mole of acetate. This was illustrated by the lower biomass yield obtained in chemostat cultivations, where no acetate accumulation was seen and therefore the effect of proton decoupling should have been minimized. The differential gene expression analysis revealed that the major transcriptional reprogramming in xfpk(BB) compared to the control was centered around the formation and metabolism of acetate. This coupled the increased oxygen demand of strain xfpk(BB) to a respiratory assimilation of acetate, which likely is the cause for the increased CO_2_ production.

Taken together, our results strongly suggest that it should be possible to circumvent the majority of the negative growth-related effects observed when phosphoketolases are expressed by minimizing acetate formation and channeling the carbon directly towards acetyl-CoA without passing through the ATP-expensive acetyl-CoA synthetase. Deletion of both yeast genes *GPP1* and *GPP2* unfortunately has strong negative effects on cell growth, which is why a *GPP1*-deletion strategy is preferable due to its higher impact on AcP hydrolysis [14, 15]. In such a genetic background, expression of xfpk(BB) in combination with a heterologous phosphotransacetylase and overexpression of acetyl-CoA consuming pathways should be able to generate an efficient carbon flux with lower negative impact on physiology. In addition to showing promise for acetyl-CoA engineering strategies, the distinct increase in E4P-levels seen when xfpk(BB) was expressed indicates that F6P-specific phosphoketolases would be beneficial for metabolic engineering strategies targeting aromatic amino acids and chemicals in yeast.

## Methods

### Strains and cultivation conditions

The strains investigated in this study were constructed previously [14]: strains AB3 and AB10, corresponding to *S. cerevisiae* strain CEN.PK 113-5D harboring plasmids pAB3 [pSP-GM1 P_*TEF1*_-*xfpk* (*B. breve*)], and pSP-GM1 [31], respectively, complementing the parental strains’ uracil auxotrophy. See a summary of strains and plasmids used in Table 2.

**Table 2:** Strains investigated in this study

Strain/name	Genotype/plasmid	Source
CEN.PK 113-5D	*MAT*a *MAL2*-*8c SUC2 ura3*-*52*/-	P. Kötter, University of Frankfurt, Germany
AB3/xfpk(BB)	CEN.PK 113-5D/pSP-GM1 P_*TEF1*_-*xfpk*(BB)	[14]
AB10/control	CEN.PK 113-5D/pSP-GM1	[14]

Cultivation of yeast strains in shake flasks and cultivation tubes were conducted in defined minimal medium consisting of 7.5 g L^−1^ (NH_4_)_2_SO_4_, 14.4 g L^−1^KH_2_PO_4_, 0.5 g L^−1^ MgSO_4_ and 20 g L^−1^ glucose. The pH was adjusted to 6.5 with 5 M KOH. Sterile solutions of trace metal and vitamin solutions, as previously described by Verduyn et al. [32], were added after autoclavation. For batch bioreactor cultivations, the concentrations of (NH_4_)_2_SO_4_ and KH_2_PO_4_ were adjusted to 5 g L^−1^ and 3 g L^−1^, respectively, and the pH was adjusted to 4, 5 or 6 depending on the experiment performed. For chemostat cultivations, glucose was used as the limiting nutrient, at a concentration of 7.5 g L^−1^ in the feed. For bioreactor cultivations, 50 μL L^−1^ antifoam (Sigma-Aldrich, St. Louis, MO, USA) was added to the media before autoclaving.

### Bioreactor cultivations and sampling

Initial precultures were prepared in 15 mL cultivation tubes using 3 mL medium and a single colony as inoculum. Each strain was grown in biological quadruplicates (i.e. separate plasmid transformants) overnight at 30 °C and 200 rpm orbital shaking. Yeast growth was monitored by optical density measurements with a spectrophotometer (Genesis20, Thermo Fisher Scientific, Waltham, MA, USA) at a wavelength of 600 nm [OD(600)]. Tube pre-cultures were used to inoculate 100 mL cultures in 500 mL unbaffled shake flasks, to an OD(600) of 0.1. When cultures reached an OD(600) of about 1, defined volumes were harvested, centrifuged for 5 min at 3000 g, and cells were resuspended in 50 mL batch bioreactor cultivation medium. The cell suspension was used to inoculate the bioreactors [DasGip Parallel Bioreactor Systems for Microbiology (Eppendorf, Hamburg, Germany)] to an initial OD of 0.025 in a volume of 600 mL. Stirring was set to 600 rpm, aeration to 36 L h^−1^ (0.1 VVM), and temperature to 30 °C. The pH was adjusted to 5.0 as standard condition, but for the specific experiment of evaluating pH-dependent strain performance it was set to either 4.0 or 6.0, using 2 M solutions of HCl and KOH. Exhaust gas flow composition was examined using a DasGip GA4 gas analyzer (Eppendorf, Hamburg, Germany). In the case of batch cultivations at pH 4 and 6, strains were allowed to grow until stationary phase, while in the case of chemostat cultivations, feeding and outflow was turned on when strains had entered an early stage ethanol phase, at a D = 0.10 h^−1^. Physiological parameters and fluxes (q) were calculated based on samples taken during exponential growth phase or steady state conditions using a cell dry weight/OD(600) conversion factor based on cell weight measurements from cells growing exponentially or during steady state conditions at pH 5. A biomass composition of CH_1.8_O_0.5_N_0.2_ was assumed [33].

Samples for monitoring OD(600) and extracellular metabolites were take throughout batch cultivations by extracting a small volume directly from the bioreactor. During chemostat cultivations, OD(600) measurements were carried out by taking samples from the outflow to verify entry to steady state. When cultures had reached steady state [indicated by stable OD(600) and CO_2_ emission profile over at least one residence time], samples for sugar phosphate analysis, RNAseq, OD(600), cell dry weight and HPLC analysis were taken as fast as possible. Samples for sugar phosphate analysis were withdrawn from the bioreactor and immediately quenched with − 40 °C methanol, cold-centrifuged, after which collected cell pellets were snap-frozen in liquid nitrogen. Samples for RNAseq were extracted from the bioreactor and directly cooled with crushed ice, centrifuged cell pellets were washed once in ice-cold PBS, re-centrifuged, after which they were snap-frozen in liquid nitrogen.

### HPLC

In order to quantify extracellular acetate, glucose, glycerol and ethanol, fermentation samples were filtered through a 0.45 μm nylon filter (VWR International AB, Stockholm, Sweden) and analysed using the HPLC system UltiMate^®^ 3000 (Dionex, Sunnyvale, CA, USA), armed with an Aminex^®^ HPX-87H ion exclusion column (Bio-Rad, Hercules, CA, USA). The system was operated at 45 °C using 5 mM H_2_SO_4_ as eluent at a flow rate of 0.6 mL/min, and metabolite detection was conducted with RI (glucose, glycerol, acetate, ethanol) and UV (pyruvate, succinate) detectors.

### LC–MS sample preparation and analysis

Approximately 20 mg (cell dry weight) of each methanol-quenched yeast sample was extracted in 1 mL cold extraction mixture consisting of chloroform:MeOH:H_2_O (1:3:1). To each sample, 1 µg of 2-deoxy-d-glucose 6-phosphate was added as internal standard. Samples were treated in a mixer mill set to a frequency 30 Hz for 3 min, with 2 tungsten carbide beads added to each tube. Obtained extracts were centrifuged at 14,000 rpm for 10 min. 250 µL of the supernatant were transferred into an LC vial followed by evaporation with a SpeedVac. For derivatization, dried samples were dissolved in 20 µL of methoxylamine and incubated on a heat block at 60 °C for 30 min. After incubation at room temperature overnight, 6 µL of 1-methylimidazole and 12 µL of propionic acid anhydride were added and heated at 37 °C for 30 min. The reaction mixture was then evaporated to dryness by N_2_ gas. Prior to LC–MS analysis, derivatized metabolites were dissolved in 100 µL of aqueous 0.1% formic acid.

Quantitative analysis was done by combined ultra-high-performance liquid chromatography-electrospray ionization-triple quadrupole-tandem mass spectrometry (UHPLC-ESI-QqQ-MS/MS) in dynamic multiple-reaction-monitoring (MRM) mode. An Agilent 6495 UHPLC chromatograph equipped with a Waters Acquity BEH 1.7 µm, 2.1 × 100 mm column (Waters Corporation, Milford, USA) coupled to a QqQ-MS/MS (Agilent Technologies, Atlanta, GA, USA) was used. The washing solution for the auto sampler syringe and injection needle was 90% MeOH with 1% HCOOH. The mobile phase consisted of A, 2% HCOOH and B, MeOH with 2% HCOOH. The gradient was 0% B for 1 min followed by linear gradients from 0.1 to 30% B from 1 to 3 min then 30–40% B from 3 to 6 min, hold at 40% B from 6 to 10 min, followed by 40–70% B from 10 to 12.5 min, hold at 70% B from 12.5 to 15 min, and thereafter 70–99% B from 15 to 17.5 min. B was held at 99% for 0.5 min, and thereafter the column was re-equilibrated to 0% B. The flow rate was 0.65 mL min^−1^ during equilibration and 0.5 mL min^−1^ during the chromatographic runs. The column was heated to 40 °C, and injection volumes were 1 μL. The mass spectrometer was operated in negative ESI mode with gas temperature 230 °C; gas flow 12 L min^−1^; nebulizer pressure 20 psi; sheath gas temperature 400 °C; sheath gas flow 12 L min^−1^; capillary voltage 4000 V (neg); nozzle voltage 500 V; iFunnel high pressure RF 150 V; iFunnel low pressure RF 60 V. The fragmentor voltage 380 V and cell acceleration voltage 5 V. For a list of MRM transitions see Additional file 1: Table S2. Data were processed using MassHunter Qualitative Analysis and Quantitative Analysis (QqQ; Agilent Technologies, Atlanta, GA, USA) and Excel (Microsoft, Redmond, Washington, USA) software.

### RNAseq sample preparation, profiling and analysis

Total RNA was extracted from cell pellets derived from bioreactor cultivations using an RNeasy Mini Kit (Qiagen, Hilden, Germany) and genomic DNA contamination was removed by treating extracted samples with TURBO DNase (Thermo Fischer Scientific). RNA integrity was confirmed using a 2100 Bioanalyzer (Agilent Technologies). Total RNA samples were processed using a TruSeq Stranded mRNA HT Sample Prep Kit (Illumina) to generate a poly-A enriched cDNA library, and samples were sequenced (paired end, 2 × 76 bp) using a NextSeq 500 (Illumina). Read pairs for each sample ranged from 3.3 to 4 million. The raw data can be downloaded from the European Nucleotide Archive with access number ERP109711. The raw reads for each sample were processed using the NGI-RNAseq Pipeline (https://github.com/SciLifeLab/NGI-RNAseq) version 1.4. The packages used in the pipeline and relevant references are summarized in Additional file 1: Table S3. The reads were mapped to the S288c reference genome (https://www.ensembl.org/Saccharomyces_cerevisiae/Info/Index) with 85.9–89.6% uniquely mapped reads. The feature counts of the four technical replicates in each biological replicate were added together. Analysis of differentially expressed genes was performed using DESeq2 [34], where the adjusted p-values were calculated by the Benjamini–Hochberg method. A complete list of the results can be found in Additional file 2. Enriched gene set analysis was performed using the platform for integrative analysis of omics (PIANO) R package [35], with log_2_ fold change and adjusted p-values from the differential gene expression analysis as input. The database Ensembl (www.ensembl.org) was used to collect GO term information.

## Additional files

**Additional file 1: Table S1.** Physiological parameters calculated for control and xfpk(BB) cultivated at pH 4 and 6. **Table S2.** MRM transitions for LC-QQQ-MS analysis. **Table S3.** Version number and references for programs used in the NGI-RNAseq pipeline. **Figure S1.** Quantification of sugar phosphates in batch phase. **Figure S2.** PCA-plot of RNA-sequencing samples. **Figure S3.** Consensus heat map of gene set analysis.

**Additional file 2.** Differential gene expression analysis data.

## Abbreviations

Xfpk: phosphoketolase
Pta: phosphotransacetylase
acetyl-CoA: acetyl coenzyme A
AcP: acetyl phosphate
G1P: glucose-1-phosphate
G6P: glucose-6-phosphate
F6P: fructose-6-phosphate
DHAP: dihydroxyacetone phosphate
GAP: glyceraldehyde-3-phosphate
3PGA: 3-phosphoglyceric acid
2PGA: 2-phosphoglyceric acid
Ru5P: ribulose-5-phosphate
R5P: ribose-5-phosphate
X5P: xylulose-5-phosphate
E4P: erythrose-4-phosphate
S7P: sedoheptulose-7-phosphate
F-1,6-BP: fructose-1,6-bisphosphate

## References

[1] Krivoruchko A, Zhang Y, Siewers V, Chen Y, Nielsen J (2015). Microbial acetyl-CoA metabolism and metabolic engineering. Metab Eng.

[2] Tang X, Feng H, Chen WN (2013). Metabolic engineering for enhanced fatty acids synthesis in *Saccharomyces cerevisiae*. Metab Eng.

[3] Feng XY, Lian JZ, Zhao HM (2015). Metabolic engineering of *Saccharomyces cerevisiae* to improve 1-hexadecanol production. Metab Eng.

[4] Zhou YJJ, Buijs NA, Zhu ZW, Qin JF, Siewers V, Nielsen J (2016). Production of fatty acid-derived oleochemicals and biofuels by synthetic yeast cell factories. Nat Commun.

[5] Rodriguez S, Denby CM, Van Vu T, Baidoo EE, Wang G, Keasling JD (2016). ATP citrate lyase mediated cytosolic acetyl-CoA biosynthesis increases mevalonate production in *Saccharomyces cerevisiae*. Microb Cell Fact.

[6] Yu T, Zhou YJ, Huang M, Liu Q, Pereira R, David F, Nielsen J (2018). Reprogramming yeast metabolism from alcoholic fermentation to lipogenesis. Cell.

[7] Kozak BU, van Rossum HM, Benjamin KR, Wu L, Daran JMG, Pronk JT, van Maris AIJA (2014). Replacement of the *Saccharomyces cerevisiae* acetyl-CoA synthetases by alternative pathways for cytosolic acetyl-CoA synthesis. Metab Eng.

[8] Zhang YM, Dai ZJ, Krivoruchko A, Chen Y, Siewers V, Nielsen J (2015). Functional pyruvate formate lyase pathway expressed with two different electron donors in *Saccharomyces cerevisiae* at aerobic growth. FEMS Yeast Res.

[9] Dai ZJ, Huang MT, Chen Y, Siewers V, Nielsen J (2018). Global rewiring of cellular metabolism renders *Saccharomyces cerevisiae* Crabtree negative. Nat Commun.

[10] Kozak BU, van Rossum HM, Luttik MA, Akeroyd M, Benjamin KR, Wu L, de Vries S, Daran JM, Pronk JT, van Maris AJ (2014). Engineering acetyl coenzyme A supply: functional expression of a bacterial pyruvate dehydrogenase complex in the cytosol of *Saccharomyces cerevisiae*. MBio.

[11] Papini M, Nookaew I, Siewers V, Nielsen J (2012). Physiological characterization of recombinant *Saccharomyces cerevisiae* expressing the *Aspergillus nidulans* phosphoketolase pathway: validation of activity through C-13-based metabolic flux analysis. App Microbiol Biotechnol.

[12] Kocharin K, Siewers V, Nielsen J (2013). Improved polyhydroxybutyrate production by *Saccharomyces cerevisiae* through the use of the phosphoketolase pathway. Biotechnol Bioeng.

[13] de Jong BW, Shi S, Siewers V, Nielsen J (2014). Improved production of fatty acid ethyl esters in *Saccharomyces cerevisiae* through up-regulation of the ethanol degradation pathway and expression of the heterologous phosphoketolase pathway. Microb Cell Fact.

[14] Bergman A, Siewers V, Nielsen J, Chen Y (2016). Functional expression and evaluation of heterologous phosphoketolases in *Saccharomyces cerevisiae*. AMB Express.

[15] Meadows AL, Hawkins KM, Tsegaye Y, Antipov E, Kim Y, Raetz L, Dahl RH, Tai A, Mahatdejkul-Meadows T, Xu L (2016). Rewriting yeast central carbon metabolism for industrial isoprenoid production. Nature.

[16] van Rossum HM, Kozak BU, Pronk JT, van Maris AJA (2016). Engineering cytosolic acetyl-coenzyme A supply in *Saccharomyces cerevisiae*: pathway stoichiometry, free-energy conservation and redox-cofactor balancing. Metab Eng.

[17] Wasylenko TM, Stephanopoulos G (2015). Metabolomic and (13)C-metabolic flux analysis of a xylose-consuming *Saccharomyces cerevisiae* strain expressing xylose isomerase. Biotechnol Bioeng.

[18] Daran-Lapujade P, Jansen MLA, Daran JM, van Gulik W, de Winde JH, Pronk JT (2004). Role of transcriptional regulation in controlling fluxes in central carbon metabolism of *Saccharomyces cerevisiae*—A chemostat culture study. J Biol Chem.

[19] Casal M, Queiros O, Talaia G, Ribas D, Paiva S, Ramos J, Sychrová H, Kschisco M (2016). Carboxylic acids plasma membrane transporters in *Saccharomyces cerevisiae*. Yeast membrane transport.

[20] Heyland J, Fu J, Blank LM (2009). Correlation between TCA cycle flux and glucose uptake rate during respiro-fermentative growth of *Saccharomyces cerevisiae*. Microbiology.

[21] Lindberg L, Santos AXS, Riezman H, Olsson L, Bettiga M (2013). Lipidomic profiling of *Saccharomyces cerevisiae* and *Zygosaccharomyces bailii* reveals critical changes in lipid composition in response to acetic acid stress. PLoS ONE.

[22] Cipollina C, ten Pierick A, Canelas AB, Seifar RM, van Maris AJA, van Dam JC, Heijnen JJ (2009). A comprehensive method for the quantification of the non-oxidative pentose phosphate pathway intermediates in *Saccharomyces cerevisiae* by GC-IDMS. J Chromatogr B Anal Technol Biomed Life Sci.

[23] Averesch NJH, Kromer JO (2018). Metabolic engineering of the shikimate pathway for production of aromatics and derived compounds—present and future strain construction strategies. Front Bioeng Biotechnol.

[24] Cornish-Bowden A (1981). Thermodynamic aspects of glycolysis. Biochem Educ.

[25] van den Berg MA, de Jong Gubbels P, Kortland CJ, van Dijken JP, Pronk JT, Steensma HY (1996). The two acetyl-coenzyme A synthetases of *Saccharomyces cerevisiae* differ with respect to kinetic properties and transcriptional regulation. J Biol Chem.

[26] Brewster NK, Val DL, Walker ME, Wallace JC (1994). Regulation of pyruvate-carboxylase Iiozyme (Pyc1, Pyc2) gene-expression in *Saccharomyces cerevisiae* during fermentative and nonfermentative growth. Arch Biochem Biophys.

[27] Colby G, Ishii Y, Tzagoloff A (1998). Suppression of sdh1 mutations by the SDH1b gene of *Saccharomyces cerevisiae*. Yeast.

[28] Fleck CB, Brock M (2009). Re-characterisation of *Saccharomyces cerevisiae* Ach1p: fungal CoA-transferases are involved in acetic acid detoxification. Fungal Genet Biol.

[29] Chen Y, Zhang YM, Siewers V, Nielsen J (2015). Ach1 is involved in shuttling mitochondrial acetyl units for cytosolic C2 provision in *Saccharomyces cerevisiae* lacking pyruvate decarboxylase. FEMS Yeast Res.

[30] van Roermund CWT, Hettema EH, van den Berg M, Tabak HF, Wanders RJA (1999). Molecular characterization of carnitine-dependent transport of acetyl-CoA from peroxisomes to mitochondria in *Saccharomyces cerevisiae* and identification of a plasma membrane carnitine transporter, Agp2p. EMBO J.

[31] Partow S, Siewers V, Bjorn S, Nielsen J, Maury J (2010). Characterization of different promoters for designing a new expression vector in *Saccharomyces cerevisiae*. Yeast.

[32] Verduyn C, Postma E, Scheffers WA, Vandijken JP (1992). Effect of benzoic-acid on metabolic fluxes in yeasts—a continuous-culture study on the regulation of respiration and alcoholic fermentation. Yeast.

[33] Villadsen I, Nielsen J, Lidén G (2011). Bioreaction engineering principles.

[34] Love MI, Huber W, Anders S (2014). Moderated estimation of fold change and dispersion for RNA-seq data with DESeq2. Genome Biol.

[35] Varemo L, Nielsen J, Nookaew I (2013). Enriching the gene set analysis of genome-wide data by incorporating directionality of gene expression and combining statistical hypotheses and methods. Nucleic Acids Res.

