# Supplementary material for: Heterologous phosphoketolase expression redirects flux towards acetate, perturbs sugar phosphate pools and increases respiratory demand in Saccharomyces cerevisiae
Source: Microb Cell Fact. 2019 Feb 1;18:25. doi: 10.1186/s12934-019-1072-6 (PMC6359841; doi:10.1186/s12934-019-1072-6)
Supplement: Supplementary file 1 — Additional file 1: Table S1. Physiological parameters calculated for control and xfpk(BB) cultivated at pH 4 and 6. Table S2. MRM transitions for LC-QQQ-MS analysis. Table S3. Version number and references for programs used in the NGI-RNAseq pipeline. Figure S1. Quantification of sugar phosphates in batch phase. Figure S2. PCA-plot of RNA-sequencing samples. Figure S3. Consensus heat map of gene set analysis. [file 12934_2019_1072_MOESM1_ESM.pdf]

Additional file 1

Supplementary tables

Table S1. Physiological parameters calculated for control and xfpk(BB) cultivated at pH 4 and 6.

	Control		xfpk(BB)	
	pH 4	pH6	pH 4	pH6
μ(max)^{Glucose} (h^{-1})	0.348 ± 0.008	0.343 ± 0.015	0.309 ± 0.001	0.298 ± 0.010
μ(max)^{EtOH} (h^{-1})	0.085 ± 0.002	0.062 ± 0.006	0.040 ± 0.003	0.070 ± 0.005
Final biomass concentration (gCDW L^{-1})	8.42 ± 0.15	6.71 ± 0.42	6.39 ± 0.01	6.72 ± 0.00
Y(x/s) (gCDW $gGlucose^{-1}$)	0.131 ± 0.001	0.133 ± 0.008	0.116 ± 0.003	0.127 ± 0.003
q(Glucose) (mmol $gCDW^{-1}$ h^{-1})	-14.73 ± 0.21	-14.76 ± 0.47	-15.9 ± 0.5	-13.03 ± 0.32
q(EtOH) (mmol $gCDW^{-1}$ h^{-1})	19.96 ± 0.61	16.7 ± 1.5	21.85 ± 1.59	16.3 ± 0.14
q(Acetate) (mmol $gCDW^{-1}$ h^{-1})	0.17 ± 0.01	0.60 ± 0.00	0.84 ± 0.02	1.61 ± 0.05
q(Glycerol) (mmol $gCDW^{-1}$ h^{-1})	0.95 ± 0.06	1.64 ± 0.17	0.76 ± 0.09	0.94 ± 0.11
q(CO_2) (mmol $gCDW^{-1}$ h^{-1})	28.86 ± 0.68	29.09 ± 1.48	34.11 ± 2.38	34.3 ± 0.56
q(Biomass) (mmol $gCDW^{-1}$ h^{-1})	14.14 ± 0.32	13.92 ± 0.62	12.56 ± 0.05	12.12 ± 0.39
Q(O_2) (mmol $gCDW^{-1}$ h^{-1})	-3.22 ± 0.41	-5.34 ± 0.49	-4.24 ± 0.26	*-3.22**
Carbon balance	97% ± 0%	92% ± 1%	98 ± 3%	108 ± 0%

Strains were cultivated in biological duplicates. Presented values represent the average ± standard deviation. Asterisk (*) indicates that only one value of q(O_2) could be calculated, as one of the O_2 detectors malfunctioned during the bioreactor run.

Table S2. MRM transitions for LC-QQQ-MS analysis. RT=retention time; CE = Collision Energy.

**Compound1**	**RT (min)**	**Quantifier**	**CE**	**Qualifier**	**CE**
2-phosphoglyceric acid	1.3	241→79	50	241→151	5
3-phosphoglyceric acid	1.55	241→79	50	241→151	5
3-phophoglyceraldehyde	3.95	254→79	50	254→180	5
Dihydroxyacetone phosphate	3.7	254→79	50	254→180	5
Erythrose-4-P	6.2	340→79	50	340→266	5
Xylulose-5-P	8.5	426→79	49	426→37	9
Ribulose-5-P	9.1	426→79	49	426→37	9
2-Deoxyglucose (IS)	9	440→79	41	-	-
Ribose-5-P	10.6	426→79	49	426→37	9
Glucose-1-P	11.8	483→79	37	483→427	13
Glucose-6-P	12.45	512→79	37	512→153	13
Fructose-6-P	12.2	512→79	37	512→153	13
Seduheptulose-7-P	13.2	598→79	49	598→542	17

Table S3. Version number and references for programs used in the NGI-RNAseq pipeline.

**Program**	**Version**	**Reference**
FastQC	0.11.7	
Trim Galore!	0.4.4	
Star	2.5.3a	Dobin A, Davis CA, Schlesinger F, Drenkow J, Zaleski C, Jha S, et al. STAR: ultrafast universal RNA-seq aligner. Bioinformatics. 2013;29(1):15–21
featureCounts	1.6.0	Liao Y, Smyth GK, Shi W. featureCounts: an efficient general purpose program for assigning sequence reads to genomic features. Bioinformatics. 2014;30(7):923–30

StringTie	1.3.3	Pertea M, Pertea GM, Antonescu CM, Chang T-C, Mendell JT, Salzberg SL. StringTie enables improved reconstruction of a transcriptome from RNA-seq reads. Nat Biotechnol. 2015;33(3):290–5
RSeQC	2.6.4	Wang L, Wang S, Li W. RSeQC: quality control of RNA-seq experiments. Bioinformatics. 2012;28(16):2184–5
dupradar	1.8.0	Sayols S, Scherzinger D, Klein H. dupRadar: a Bioconductor package for the assessment of PCR artifacts in RNA-Seq data. BMC Bioinformatics. 2016;17(1):428.
Preseq	2.0.1	Daley T, Smith AD. Predicting the molecular complexity of sequencing libraries. Nat Methods. 2013;10(4):325–7.
Picard MarkDuplicates	2.18.0	
edgeR	3.20.1	Robinson MD, McCarthy DJ, Smyth GK. edgeR: a Bioconductor package for differential expression analysis of digital gene expression data. Bioinformatics. 2010;26(1):139–40
MultiQC	1.5	Ewels P, Magnusson M, Lundin S, Käller M. MultiQC: summarize analysis results for multiple tools and samples in a single report. Bioinformatics. 2016;32(19):3047–8
nextflow	0.29.0	Di Tommaso P, Chatzou M, Floden EW, Barja PP, Palumbo E, Notredame C. Nextflow enables reproducible computational workflows. Nat Biotechnol. 2017;35(4):316–9

Supplementary figures

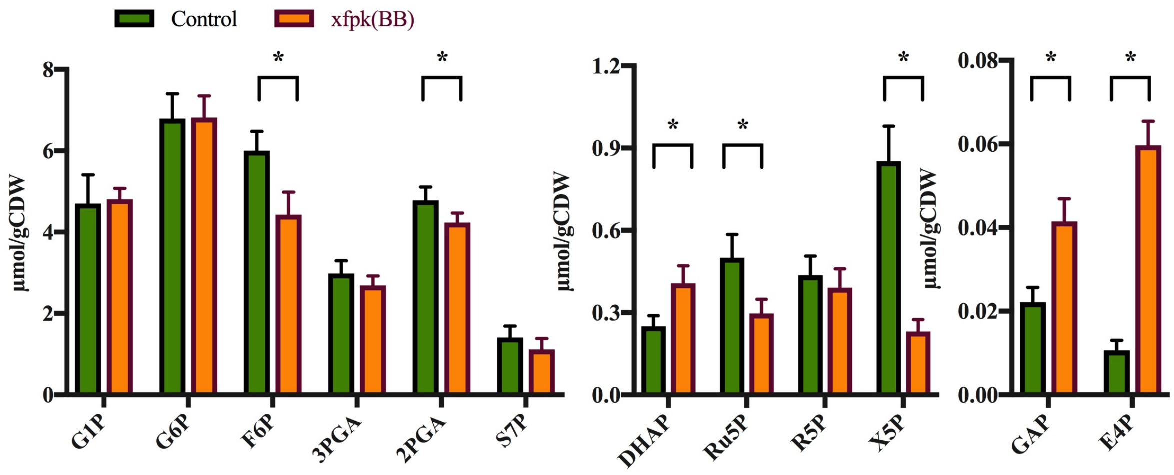

Figure S1. Quantification of sugar phosphates in batch phase. Strains were grown in 2% glucose in biological quadruplicates (samples taken in exponential growth phase). Significant changes ($p<0.05$, Students t-test, two-sided, unequal variance assumed) are indiciated with asterisks (*), error bars equals ± standard deviation.

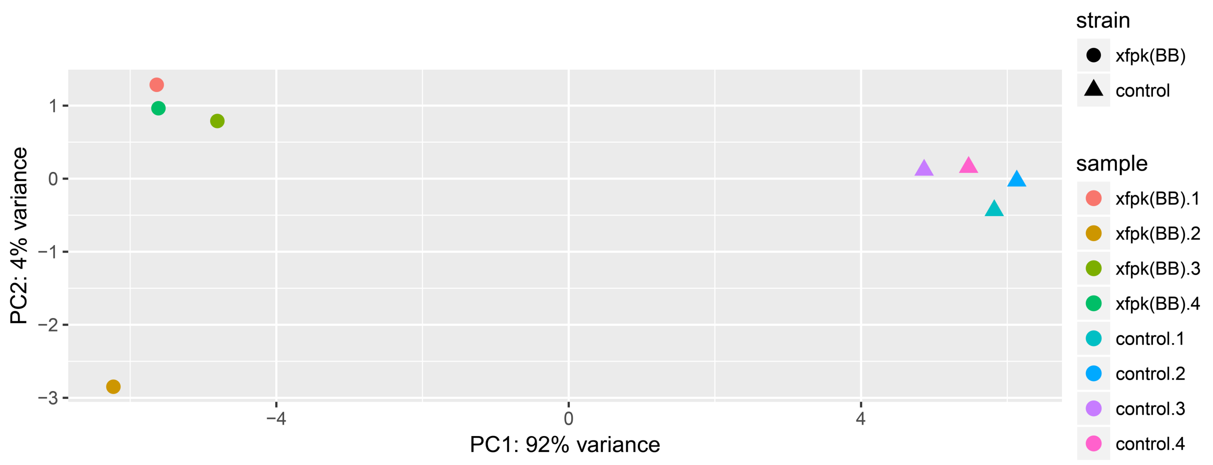

Figure S2. PCA-plot of RNA-sequencing samples.

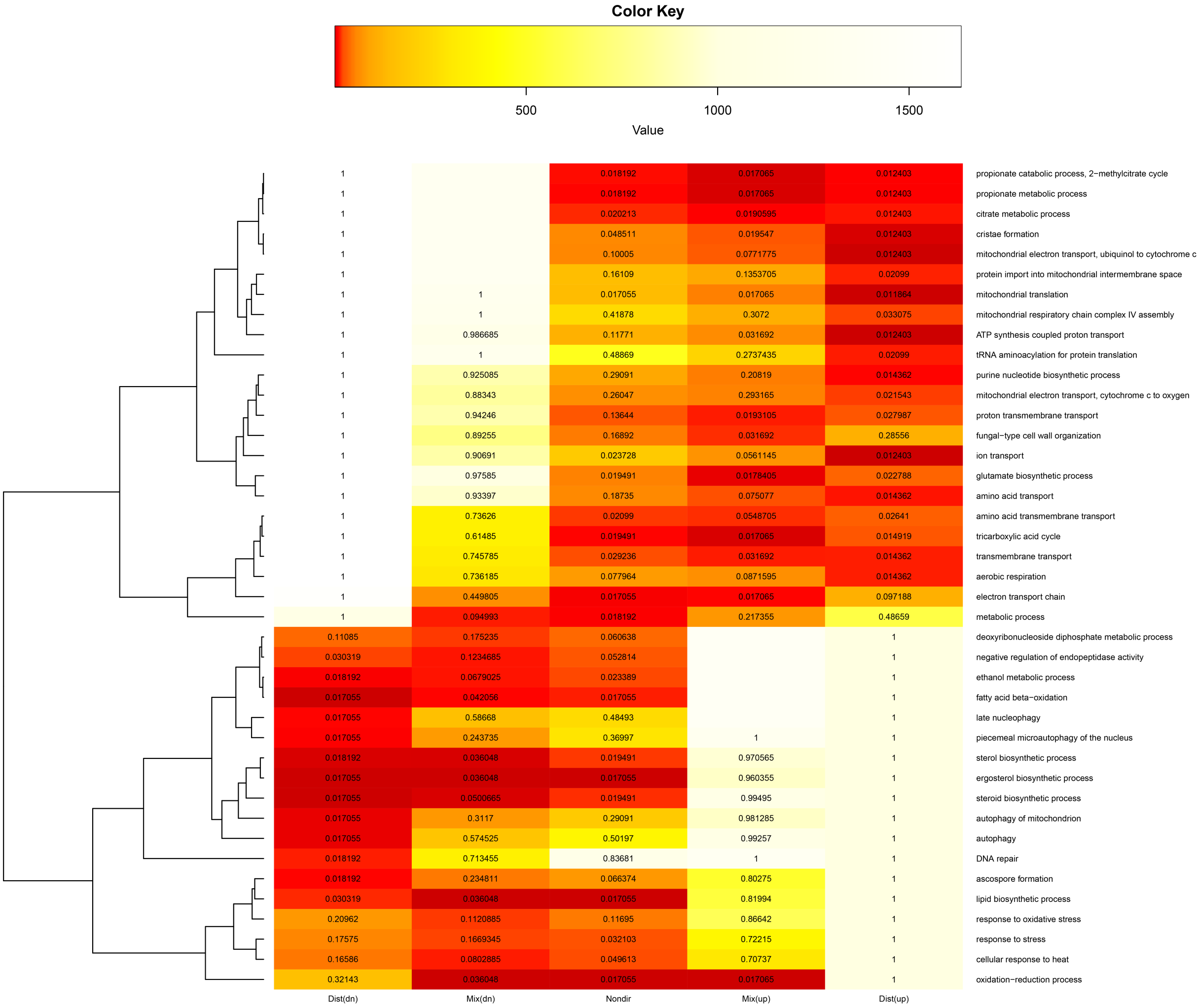

Figure S3. Consensus heat map of gene set analysis. xfpk(BB) overexpression strain is compared to the control during chemostat cultivation. The number in each cell is the median adjusted p-value from different GSA methods (mean, median, sum, mazmean, fisher, stouffer, tailStrength, gsea and page). The color key is the consensus rank (mean rank of each gene set from each GSA method). See "Varemo L, Nielsen J, Nookaew I. Enriching the gene set analysis of genome-wide data by incorporating directionality of gene expression and combining statistical hypotheses and methods. Nucleic Acids Res. 2013;41(8):4378-91" for details.
